# Type 2 Diabetic Rats on Diet Supplemented With Chromium Malate Show Improved Glycometabolism, Glycometabolism-Related Enzyme Levels and Lipid Metabolism

**DOI:** 10.1371/journal.pone.0125952

**Published:** 2015-05-05

**Authors:** Weiwei Feng, Ting Zhao, Guanghua Mao, Wei Wang, Yun Feng, Fang Li, Daheng Zheng, Huiyu Wu, Dun Jin, Liuqing Yang, Xiangyang Wu

**Affiliations:** 1 School of Food and Biological Engineering, Jiangsu University, Zhenjiang, Jiangsu, China; 2 School of Chemistry and Chemical Engineering, Jiangsu University, Zhenjiang, Jiangsu, China; 3 School of the Environment and Safety Engineering, Jiangsu University, Zhenjiang, Jiangsu, China; 4 School of Medical Science and Laboratory Medicine, Jiangsu University, Zhenjiang, Jiangsu, China; 5 School of Pharmacy, Jiangsu University, Zhenjiang, Jiangsu, China; University of Catanzaro Magna Graecia, ITALY

## Abstract

Our previous study showed that chromium malate improved the regulation of blood glucose in mice with alloxan-induced diabetes. The present study was designed to evaluate the effect of chromium malate on glycometabolism, glycometabolism-related enzymes and lipid metabolism in type 2 diabetic rats. Our results showed that fasting blood glucose, serum insulin level, insulin resistance index and C-peptide level in the high dose group had a significant downward trend when compared with the model group, chromium picolinate group and chromium trichloride group. The hepatic glycogen, glucose-6-phosphate dehydrogenase, glucokinase, Glut4, phosphor-AMPKβ1 and Akt levels in the high dose group were significantly higher than those of the model, chromium picolinate and chromium trichloride groups. Chromium malate in a high dose group can significantly increase high density lipoprotein cholesterol level while decreasing the total cholesterol, low density lipoprotein cholesterol and triglyceride levels when compared with chromium picolinate and chromium trichloride. The serum chromium content in chromium malate and chromium picolinate group is significantly higher than that of the chromium trichloride group. The results indicated that the curative effects of chromium malate on glycometabolism, glycometabolism-related enzymes and lipid metabolism changes are better than those of chromium picolinate and chromium trichloride. Chromium malate contributes to glucose uptake and transport in order to improved glycometabolism and glycometabolism-related enzymes.

## Introduction

Type 2 diabetes (T2D), which appears after age 40 and accounts for more than 90% of diabetes cases, is a serious public health problem [1,2]. T2D is a problem found worldwide, including in China. Previous studies have shown that diet, as well as genetic and environmental parameters, can induce T2D [3–6]. Moreover, T2D is implicated in disorders of glycometabolism, glycometabolism-related enzymes, and lipid metabolism. Furthermore, T2D is associated with complications such as learning and memory deficits, changes in intestinal flora, diabetic nephropathy, diabetic peripheral neuropathy and psychological distress. Suge et al [7] have reported that T2D can induce impairment in brain function, resulting in learning deficits. Michelle et al [8] reported that subjects with T2D performed significantly lower than healthy subjects on tests of cognitive ability. Xu et al [9] have reported that T2D patients also exhibit changes in intestinal flora. These complications can negatively affect patients’ health and quality of life, especially amongst ethnic minorities and populations living in remote areas [10]. Currently, medical treatment is an effective means of managing T2D [11]. Some drugs, including biguanides, sulfonylureas, insulin and traditional Chinese medicines, can maintain blood sugar levels by reducing hepatic glycogen output, stimulating insulin secretion or increasing insulin sensitivity, respectively. However, diabetes and diabetes-related complications can not be cured. Treatment of T2D cost the United States $245 billion in 2012, including $176 billion in direct medical costs [12]. Pharmacological treatment cannot always prevent or alleviate diabetes-related complications. However, studies have shown that a healthy diet and exercise can also be effective in managing T2D [13,14].

Chromium (Cr) occurs in the environment primarily in two common oxidation states: Cr(III) and Cr(VI) [15]. It is a beneficial micronutrient and has shown significant anti-diabetic activity [16–18]. Insulin level and sensitivity drop when the body lacks sufficient chromium, resulting in disorders of glucose and lipid metabolism. Studies have found that Cr can regulate lipid metabolism and enhance insulin sensitivity [19–21]. However, inorganic chromium compounds such as chromium trichloride have some drawbacks, including low absorption rate and toxicity [22,23]. Organic chromium complexes, including chromium picolinate, chromium nicotinate and chromium yeast, have been widely used as functional food ingredients and nutritional supplements. To date, chromium picolinate is the organic complex most commonly utilized as a dietary supplement. However, it has been reported that ingestion of chromium picolinate can result in genotoxicity and cytotoxicity thought to be caused by the ligand (picolinate), thus raising questions regarding the compound’s safety [24–26]. Use of chromium nicotinate and chromium yeast as supplements is not widespread due to poor solubility and unstable structure, respectively. Therefore, characterizing the anti-hyperglycemic activity of novel and non-toxic organic chromium complexes is an important problem.

Chromium malate is a new type of organic chromium compound that has been synthesized in our research group [27]. Cr^3+^ was chelated by L-malic acid, which was selected as the natural ligand. Our previous study showed that chromium malate improved the regulation of blood glucose in mice with alloxan-induced diabetes. Chromium malate does not cause oxidative DNA damage and was tested as non-toxic in acute and subacute toxicity studies [28]. Therefore, chromium malate has potential for application as a functional food and nutrient supplement for the improvement of glycometabolism, production of glycometabolism-related enzymes and lipid metabolism. However, few studies on this use of chromium malate exist. In the present study, chromium malate was given to type 2 diabetic rats, and their glycometabolism, glycometabolism-related enzymes and lipid metabolism were evaluated. The protective effect of chromium malate on learning and memory ability, related enzymes and intestinal flora were studied. This study will provide a basis for research and development of a safe, non-toxic organic chromium complex with a high absorption rate.

## Methods

### Ethics statement

All the experimental procedures were conducted in accordance with The Code of Ethics of the World Medical Association (Declaration of Helsinki) for experiments involving humans, EC Directive 86/609/EEC for animal experiments and approved by the Jiangsu University Committee on Animal Care and Use(the license number SYXK (SU) 2013–0036). Sprague-Dawley rats were procured from the Jiangsu University. Sprague-Dawley rat is not a protected nor endangered species. Our experiments complied with the laws and ethical recommendations currently in effect in China where the experiments were performed. The rats were anaesthetized by anhydrous diethyl ether in order to ameliorate suffering. Then the rats were sacrificed at unconscious situation.

### Materials and chemicals

The chromium (III) malate was synthesized according to the method described by Li et al. [28] in our research group. Chromium picolinate and chromium trichloride were obtained from Sinopharm Chemical Reagent Co., Ltd (Shanghai, China). Cholesterol and sodium cholate hydrate were produced at Hubei Hongyun long biological technology Co. Ltd. and Henan zhengxing food additive co., Ltd, respectively. Streptozotocin(STZ) was obtained from Sigma Chemical Co. Distilled water was used throughout the whole experiments.

### Experimental animals and diets

Male Sprague-Dawley rats (180±10g) were procured from the Jiangsu University for the experiment. The distilled water was supplied during the entire experimental period. The temperatures of the rats house and relative humidity were 24±1°C and 55–60%, respectively.

### Study design and Models of T2D

The rats were randomly divided into eight groups of ten animals. In the first two months, high sugar and high fat diet was supplied to rats. The main components of the feed are standard rat pellet diet(78.25%), lard(10%), sucrose(10%), Cholesterol(1.5%) and Sodium cholate hydrate(0.25). STZ was dissolved in citrate buffer at pH 4.5. Rats were injected with STZ through intraperitoneal injection at a dose of 30 mg/kg body weight. Following the injection, standard rat pellet diet and distilled water were available during the remaining 4 weeks of the experiment. The equal volume of 0.9% saline was given to normal rats. Three days later, fasting blood glucose (FBG) levels were measured by one touch glucometer. Blood sample was drawn from the tail vein of rats. At this point, the oral glucose tolerance test and insulin resistance test were conducted. The FBG levels of rats were 33.3mmol/L > FBG ≥ 11.1 mmol/L, accompanied by oral glucose tolerance and insulin resistance reduced were taken as successful induction of T2D.

### Anti-hyperglycemic Activity of chromium malate

The experiment design and the dose of chromium malate, chromium picolinate and chromium trichloride were shown in Table 1. The dosage of chromium malate to rats was calculated with chromium. The type 2 diabetic rats were treated with chromium malate once per day for 4 weeks, the FBG level and body mass of rats were tested once a week.

**Table 1:** The experiment design and the dose of chromium malate_,_ chromium picolinate and chromium trichloride.

Group	Rats	Dose (Cr, μg/kg bw)
**Normal control group**	Normal rats	0
**Model group**	Type 2 diabetic rats	0
**Chromium picolinate group**	Type 2 diabetic rats	20.0
**Chromium trichloride group**	Type 2 diabetic rats	20.0
**Chromium malate group (Low dose group)**	Type 2 diabetic rats	10.0
**Chromium malate group (Middle dose group)**	Type 2 diabetic rats	15.0
**Chromium malate group (High dose group)**	Type 2 diabetic rats	20.0
**Chromium malate control group**	Normal rats	20.0

### Glycometabolism and glycometabolism-related enzymes study

After the rats were sacrificed on the 29 days, the serum and liver were collected. Rat ELISA kits were used to assay serum insulin and C-peptide (C-P) in the samples of rat^,^s blood serum. Insulin resistance index (IR) was calculated by this formula.

$$IR=\;Insulin/22.5e^{-lnFBG}$$ (1)

Hepatic glycogen level was estimated according to the glycogen assay kit (Nanjing Jiancheng biological technology Co. Ltd.). The liver was homogenized in ice-cold physiological saline at the same time. The homogenate was centrifuged at 10000 g for 10 min, and then collected supernatant. Glucose-6-phosphate dehydrogenase (G6PD) and glucokinase (GCK) in the liver supernatant were assayed by rat ELISA kits.

### Glucose uptake and transport study

BRL rat hepatic cell was maintained at 37°C in a humidified atmosphere of 5% CO_2_ and grown in DMEM-high glucose supplemented with 10% foetal bovine serum. The BRL rat hepatic cell was incubated with 1*10^–6^ mol/L insulin for 24 h and then treated with chromium malate, chromium picolinate and chromium trichloride for 24 h. Protein was extracted and determined using the total protein extraction kit and BCA proteinassay kit, respectively. Western blot was used to analysis the target protein(Glut4, phosphor-AMPKβ1 and Akt). The bands of the target protein were scanned, and the relative density of each band was calculated with Quantity One software. The relative density of the target protein was normalized to the density of GAPDH.

### Lipid metabolism study

The serum, which was collected on the 29 days, was used to measure lipid content. Total cholesterol (TC), low density lipoprotein cholesterol (LDL), high density lipoprotein cholesterol (HDL) and triglyceride (TG) levels in blood serum were measured by enzymatic colorimetric method using a reagent kit (Dong’ou Jinma Technology, Co., Ltd., Zhejiang, China). Blood serum (0.1 mL) was transferred to the tube containing operating fluid, and the mixture was heated at 37°C for 5 min. The sample was measured by its absorption at 546 nm.

### Cr content analysis

The serum and organ (heart, liver, spleen, lung, kidney, brain) of rats, which were collected in above experiment, were used to measure Cr content. Literatures have reported that ICP method can be used for determination of Cr in rat serum and organs [27,29]. Vista-MPX Simultaneous Inductively coupled plasma (ICP) method (Varian, Inc. USA) was used to determination of Cr content.

### Histopathological analyses

The rats’ liver and kidney were undergone hematoxylin and eosin staining, and then examined macroscopically. The methods of staining were estimated according to the method of Wu et al. [27]. The morphology of the liver and kidney were visually observed for signs of toxicity.

### The organ index analysis

The heart, liver, spleen, lung, kidney, brain and hippocampus were collected and weighed when the rats were sacrificed. Relative heart/liver/spleen/lung/kidney/brain weights were expressed as the ratio of the heart/liver/spleen/lung/kidney/brain to body weight (mg/g).

### The hematological and biochemical analysis

Blood samples of normal rats and type 2 diabetic rats were collected through retro-orbital puncture on the 29 days. The blood samples were centrifuged at 10000 g for 10 min in order to get blood serum. Then the anti-coagulated blood and serum were used for hematological and biochemical analyses.

### Learning and memory ability and related enzymes study

#### Morris water maze test

The Morris water maze test was used to evaluate the spatial learning abilities of rats and perform using the method of Enomoto et al. [30] with slight modification. At the end of the experimental period, rats were trained for 3 trials per day for 4 days. The training time was 120 s. The rats’ escape latency and swimming speed to arrive the platform were measured. The space exploration experiment was carried out at the same time.

#### The related enzyme analysis

The hippocampus of rat was collected after the rats were sacrificed. The hippocampus was homogenized in ice-cold physiological saline. The homogenate was centrifuged at 10000 g for 10 min, and then the supernatant was collected. The superoxide dismutase (SOD), glutathione peroxidase (GSH-PX) and acetylcholinesterase (AChE) in supernatant were analyzed by UV—2450 UV-vis spectrophotometer (SHIMADZU, Japan).

### Effect of chromium malate on intestinal flora changes of rats

On the last day of the experiment, the faeces of rats were collected at aseptic conditions. The gram staining was used to detect the number of intestinal flora in normal rats and type 2 diabetic rats. The smear was immobilized by fired, and then crystal violet dye solution was used to staining for 1 minute. Then the iodine liquid, decoloring liquid and after dyeing liquid were used to staining in turn. The gram-positive bacteria (G^+^b), gram-negative bacteria (G^-^b), gram-positive cocci (G^+^c) and gram-negative cocci (G^-^c) were examined under the microscope (10 × 40 times, 200 bacterial).

### Statistical analysis

Statistical analyses were performed with the program SPSS 16.0. Averages and standard error of the mean were expressed as mean ± SEM number of observations. The one-way analysis of variance (ANOVA) was used for data analysis. The Tukey test for the multiple comparisons among the groups was performed to determine the significant differences. A value of P < 0.05 among groups was considered statistically significant.

## Results and Discussion

### Models of T2D

The FBG levels of STZ-induced type 2 diabetic rats were 33.3 mmol/L > FBG ≥ 11.1 mmol/L. The change in FBG levels of normal rats and T2D rats following oral gavage of glucose was monitored (S1 Fig). STZ-induced T2D rats exhibited significant hyperglycemia. As shown in S1B Fig, the AUC_0–3h_ of FBG in type 2 diabetic rats was significantly higher than that of normal rats at 1159.50±103.24 and 4722.00±292.55 for T2D and normal rats, respectively. The insulin levels and insulin resistance (IR) of type 2 diabetic rats were significantly higher than those of normal rats. Consistent with our results, Kim et al [31] have reported that rats with diabetes exhibit decreased insulin sensitivity. IR of type 2 diabetic rats and normal rats were 0.81±0.05 and 3.74±0.43, respectively. These data were consistent with results published by Salido et al [32]. In other experiments, plasma glucose levels were assessed in non-fasted animals [33]. But the FBG levels of rats were 33.3mmol/L > FBG ≥ 11.1 mmol/L, accompanied by oral glucose tolerance and insulin resistance reduced. Together, these results confirmed that the rat model of type 2 diabetes is a success.

### Anti-hyperglycemic activity of chromium malate

**Fig 1:**
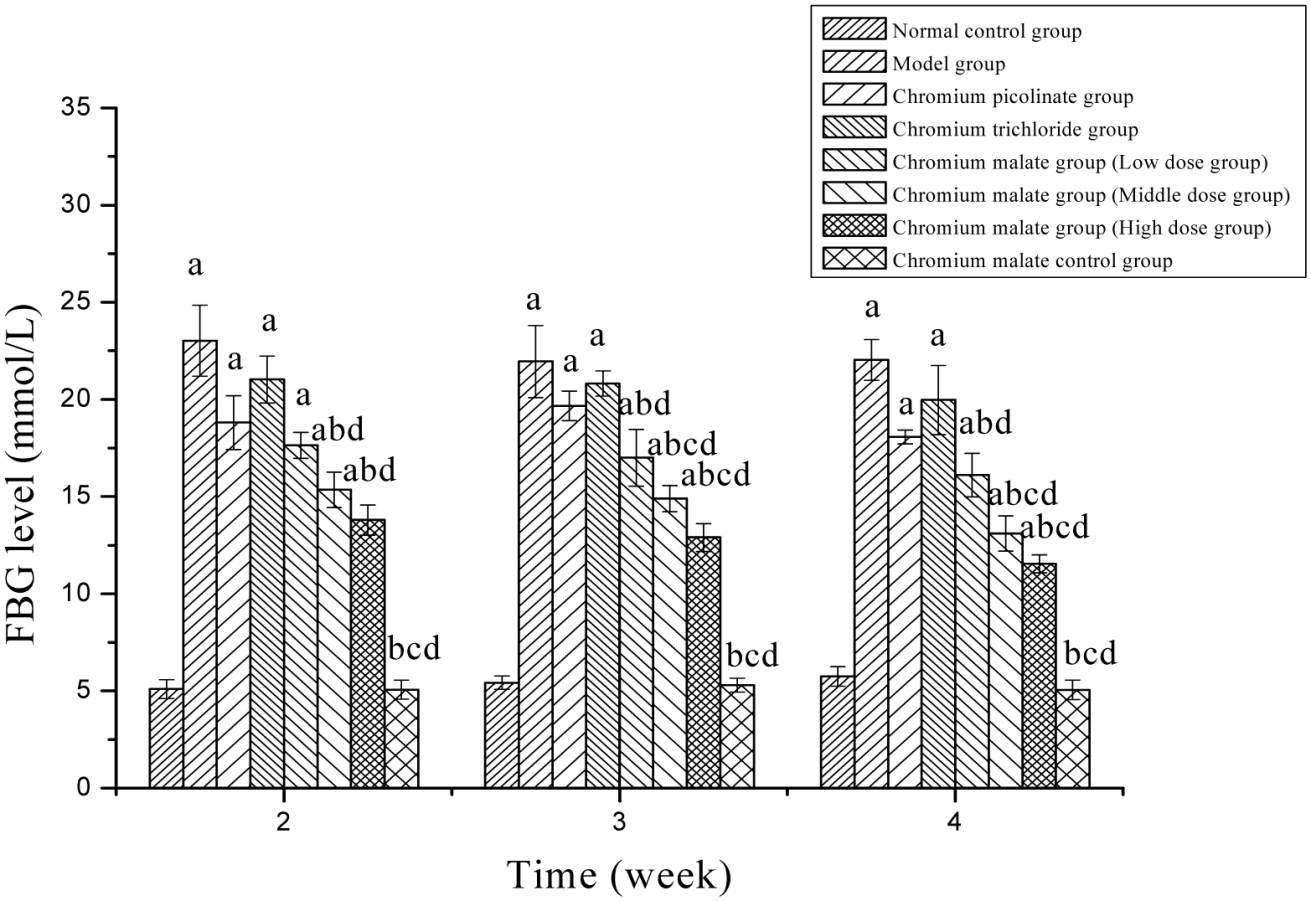
The fasting blood glucose (FBG) level (mmol/L) changes of normal and type 2 diabetic rats were treated with chromium malate. Chromium picolinate and chromium trichloride was used as a positive control. Each value was presented as means±SD (n = 10). ^a^ Significantly different from normal control group (P < 0.05). ^b^ Significantly different from model group (P < 0.05). ^c^ Significantly different from chromium picolinate group (P < 0.05). ^d^ Significantly different from chromium trichloride group (P < 0.05).

To investigate the anti-hyperglycemic activity of chromium malate on T2D, type 2 diabetic rats were examined on the 2nd, 3rd and 4th weeks following administration of chromium malate (Fig 1). Four weeks after administration of chromium malate, FBG levels had declined and remained at approximately 11.54 mmol/L (at a dose of 20.0 μg Cr /kg bw) close to FBG levels of normal rats (5.56–11.11 mmol/L) [34]. FBG levels differed significantly between the chromium malate treated group and model group. The anti-hyperglycemic activity of chromium malate exhibits a dose dependency. The FBG levels of rats treated with 10, 15 and 20 μg Cr/day/kg bw were 16.23, 13.10 and 11.54 mmol/L, respectively. Additionally, the results showed that FBG level decreased significantly with extended time of feeding with chromium malate. The changes in FBG levels of type 2 diabetic rats following oral gavage of chromium malate, chromium picolinate and chromium trichloride were compared in this study. The results confirmed that the anti-hyperglycemic activity of chromium malate (at a dose of 20.0 μg Cr/kg bw) as indicated by FBG levels was superior to that of chromium picolinate (18.07 mmol/L, at a dose of 20.0 μg Cr/kg bw) and chromium trichloride (20.25 mmol/L, at a dose of 20.0 μg Cr/kg bw). The FBG levels of rats in the chromium malate middle and high dose groups decreased significantly when compared with rats in the chromium picolinate and chromium trichloride groups. The anti-hyperglycemic activity of chromium malate is better than that of chromium picolinate and chromium trichloride. Moreover, chromium malate has no obvious effect on FBG level of normal rats.

### Glycometabolism and glycometabolism-related enzymes study

#### Effects of chromium malate on the serum insulin and IR of rats

**Fig 2:**
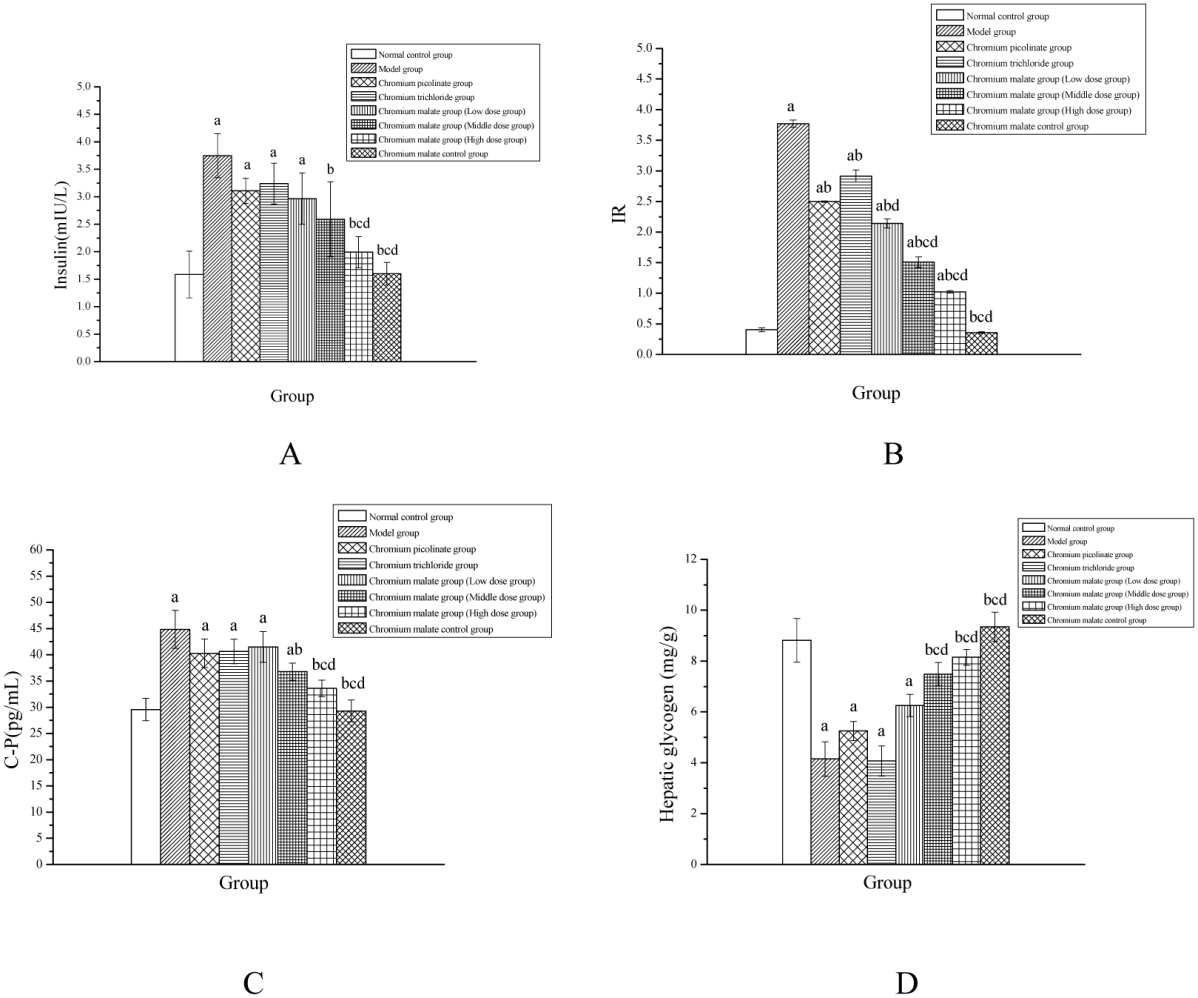
Effects of chromium malate on the serum insulin (A), insulin resistance index (IR) (B), serum C-P (C) and hepatic glycogen content changes (D) changes of normal rats and type 2 diabetic rats. Chromium picolinate and chromium trichloride was used as a positive control. Each value was presented as means±SD (n = 10). ^a^ Significantly different from normal control group (P < 0.05). ^b^ Significantly different from model group (P < 0.05). ^c^ Significantly different from chromium picolinate group (P < 0.05). ^d^ Significantly different from chromium trichloride group (P < 0.05).

The changes in serum insulin and IR of normal rats and type 2 diabetic rats following administration of chromium malate, chromium picolinate and chromium trichloride by oral gavage are shown in Fig 2A and 2B. From these results, it was deduced that chromium malate, chromium picolinate and chromium trichloride can reduce serum insulin and IR levels. The IR level of type 2 diabetic rats treated with chromium picolinate and chromium trichloride decreased significantly when compared with type 2 diabetic rats in the model group, while serum insulin levels remained comparable. In addition, the serum insulin and IR levels of type 2 diabetic rats decreased with administration of chromium malate in a dose-dependent manner. The serum insulin and IR levels of type 2 diabetic rats in the high dose group were most significantly decreased when compared with the model, chromium picolinate and chromium trichloride groups. Moreover, administration of chromium malate has no obvious effect on serum insulin and IR levels of normal rats.

#### Effects of chromium malate on the serum C-P changes of rats

C-P is secreted by pancreatic β cells, and its total amount is equivalent with that of insulin. It is an important index for detection of insulin. The serum C-P changes of normal rats and type 2 diabetic rats following administration of chromium malate, chromium picolinate and chromium trichloride by an oral gavage are shown in Fig 2C. It can be observed that chromium malate, chromium picolinate and chromium trichloride can reduce serum C-P level. However, the C-P levels of the chromium picolinate and chromium trichloride groups showed no significant decrease when compared with the model group. It can be observed that the C-P level of type 2 diabetic rats is significantly higher than that of normal rats. The serum C-P levels show a decreasing trend after administration of chromium malate. The serum C-P level of the high dose group was significantly decreased when compared with the model, chromium picolinate and chromium trichloride groups. Additionally, chromium malate has no obvious effect on the serum C-P level of normal rats. The curative effects of chromium malate on C-P changes are better than those of chromium picolinate and chromium trichloride.

#### Effects of chromium malate on hepatic glycogen levels in rats

Hepatic glycogen is composed of glucose and is stored in the liver. Humans with diabetes often exhibit low levels of hepatic glycogen. The changes in the hepatic glycogen content of rats treated with chromium malate, chromium picolinate and chromium trichloride are shown in Fig 2D. It can be observed that chromium malate and chromium picolinate can increase the content of hepatic glycogen, while chromium trichloride causes no such increase. The hepatic glycogen content of type 2 diabetic rats in the chromium picolinate group showed no significant increase when compared with model group. The results showed that the amount of hepatic glycogen in type 2 diabetic rats is significantly lower than in normal rats. The middle dose and high dose groups showed a significant increase in the amount of hepatic glycogen when compared with the chromium picolinate and chromium trichloride groups. Therefore, the curative effect of chromium malate on increased hepatic glycogen was better than that of chromium picolinate and chromium trichloride.

#### Effects of chromium malate on G6PD and GCK levels in rats

**Fig 3:**
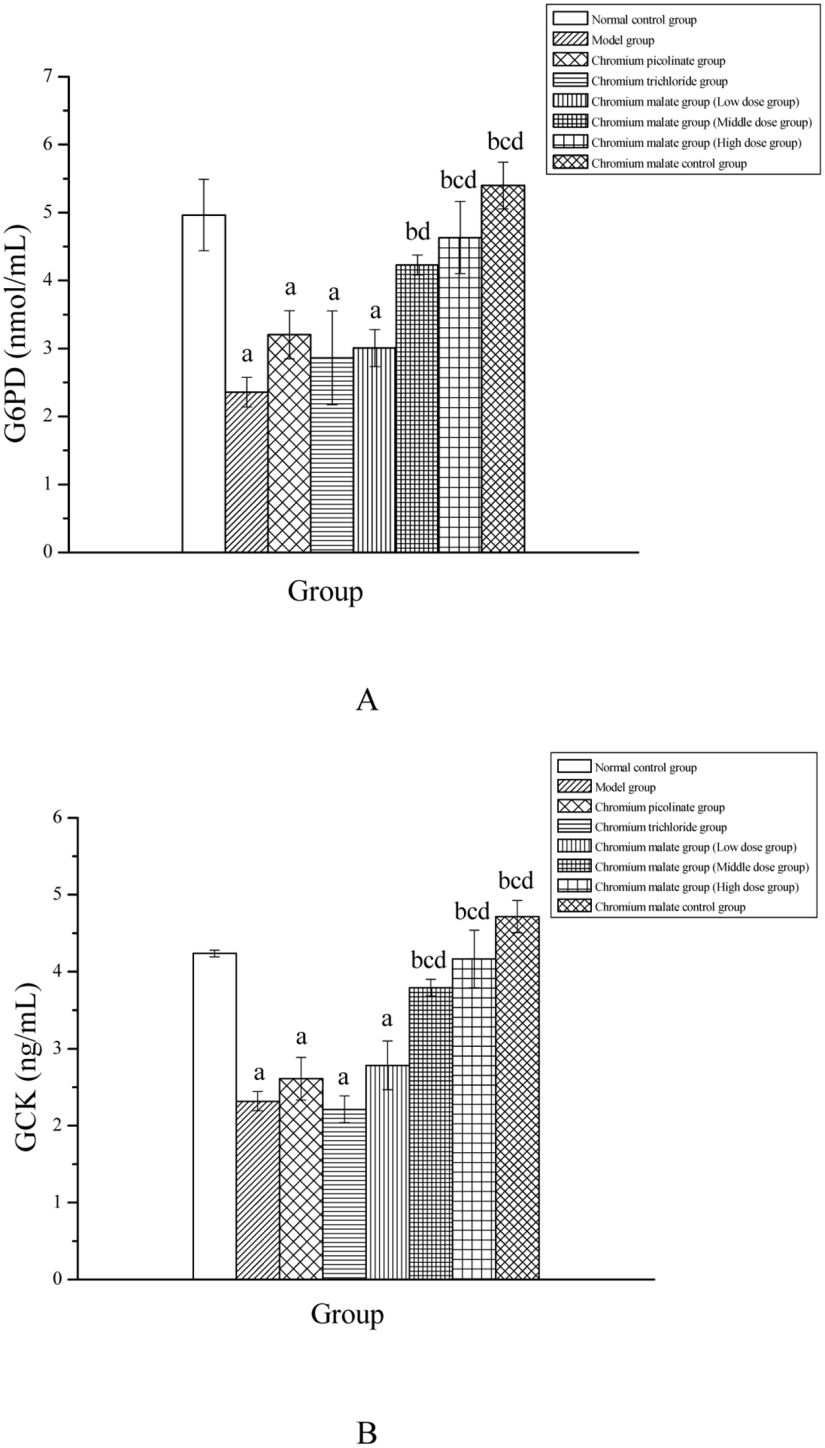
Effects of chromium malate on the Glucose-6-phosphate dehydrogenase (G6PD) (A) and Glucokinase (GCK) (B) content changes of normal rats and type 2 diabetic rats. Chromium picolinate and chromium trichloride was used as a positive control. Each value was presented as means±SD (n = 10). ^a^ Significantly different from normal control group (P < 0.05). ^b^ Significantly different from model group (P < 0.05). ^c^ Significantly different from chromium picolinate group (P < 0.05). ^d^ Significantly different from chromium trichloride group (P < 0.05).

G6PD and GCK are key enzymes in glucose metabolism found in the liver. Changes in G6PD and GCK levels in chromium malate, chromium picolinate and chromium trichloride treated rats are shown in Fig 3. Chromium malate, chromium picolinate and chromium trichloride can increase the content of G6PD (Fig 3A). However, the G6PD content of type 2 diabetic rats treated with chromium picolinate and chromium trichloride did not increase significantly when compared with model group. The results showed that the amount of G6PD in type 2 diabetic rats is significantly lower than that in normal rats. Chromium malate can significantly increase the content of G6PD in the middle and high dose groups when compared with model group. The G6PD content of type 2 diabetic rats in the high dose group is significantly higher than that of chromium picolinate and chromium trichloride groups. Results for GCK content are similar to those obtained for G6PD (Fig 3B). The content of GCK in the middle and high dose groups is significantly increased compared to the model, chromium picolinate and chromium trichloride groups. In conclusion, chromium malate is more effective at treating increased G6PD and GCK content than chromium picolinate and chromium trichloride.

### Glucose uptake and transport study

**Fig 4:**
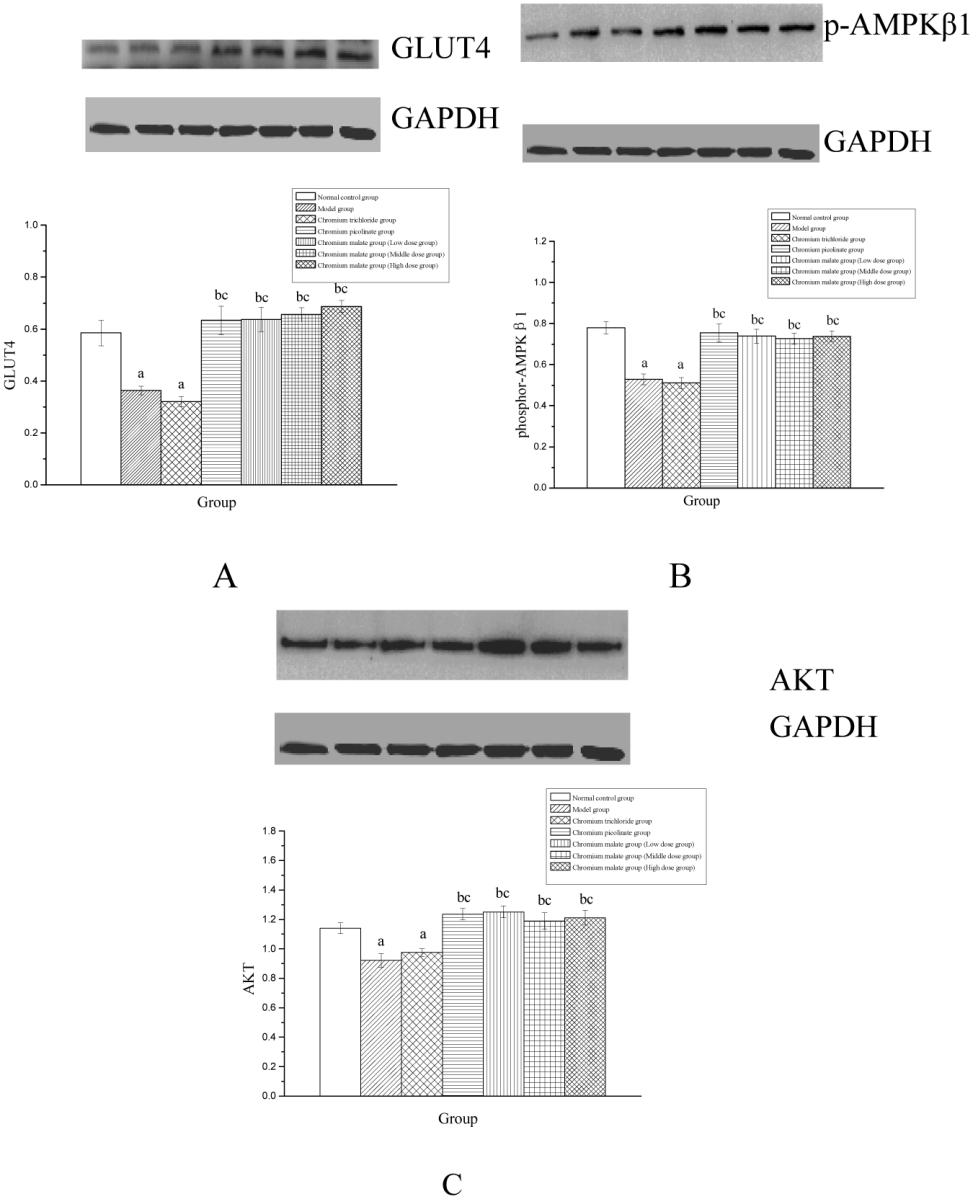
The mechanism of chromium malate to glucose uptake and transport in BRL rat hepatic cell with insulin resistance. ^a^ Significantly different from normal control group (P < 0.05). ^b^ Significantly different from model group (P < 0.05). ^c^ Significantly different from chromium trichloride group (P < 0.05). ^d^ Significantly different from chromium picolinate group (P < 0.05).

The mechanism of chromium malate to glucose uptake and transport in BRL rat hepatic cell with insulin resistance is shown in Fig 4. The results indicated that chromium malate (at a dose of 5, 10 and 20.0 μg Cr/mL) can significant increase Glut4, phosphor-AMPKβ1 and Akt levels. The Glut4, phosphor-AMPKβ1 and Akt levels of chromium malate group are significantly higher than those of BRL rat hepatic cell with insulin resistance in the model and chromium trichloride groups. The Glut4, phosphor-AMPKβ1 and Akt levels of chromium malate (at a dose of 20.0 μg Cr/mL) group are same as chromium picolinate group. In conclusion, Chromium malate contributes to glucose uptake and transport in order to improved glycometabolism and glycometabolism-related enzymes. And chromium malate is more effective at treating increased Glut4, Akt and phosphor-AMPKβ1 levels than chromium trichloride.

### Lipid metabolism study

**Fig 5:**
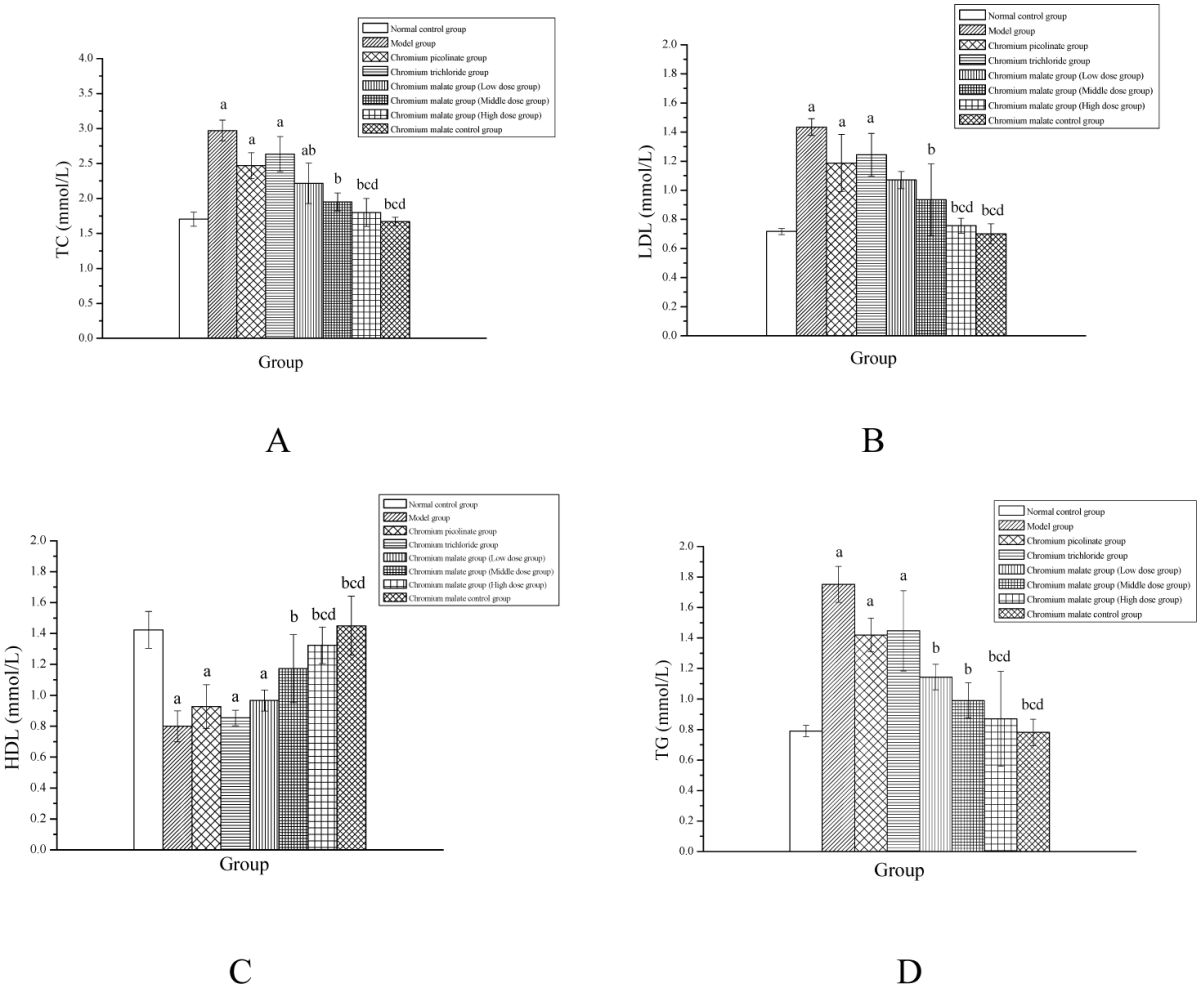
The total cholesterol (TC) (A), low density lipoprotein cholesterol (LDL) (B), high density lipoprotein cholesterol (HDL) (C) and triglyceride (TG) (D) level changes of normal rats and type 2 diabetic rats were treated with chromium malate. Chromium picolinate and chromium trichloride was used as a positive control. Each value was presented as means±SD (n = 10). ^a^ Significantly different from normal control group (P < 0.05). ^b^ Significantly different from model group (P < 0.05). ^c^ Significantly different from chromium picolinate group (P < 0.05). ^d^ Significantly different from chromium trichloride group (P < 0.05).

The serum lipids mainly include TC, LDL, HDL and TG. The ability of chromium malate to alter serum TC, LDL, HDL and TG levels in normal rats and type 2 diabetic rats is shown in Fig 5. The results indicated that chromium malate can reduce serum TC, LDL and TG levels in type 2 diabetic rats. The serum TC, LDL and TG levels of type 2 diabetic rats in the high dose group were significantly lower than those of type 2 diabetic rats in the model, chromium picolinate and chromium trichloride groups. The results also revealed that the reduction of TC, LDL and TG levels in type 2 diabetic rats by chromium malate is dose dependent. While the levels of serum TC, LDL and TG in the chromium picolinate and chromium trichloride groups were lower than those in the model group, the difference was not significant. As shown in Fig 5C, chromium malate can increase serum HDL level in type 2 diabetic rats. The HDL level of the high dose group was significantly higher than that of the model, chromium picolinate and chromium trichloride groups. However, the chromium picolinate and chromium trichloride groups did not show a significantly increased HDL level. The results confirmed that chromium malate was more effective than chromium picolinate and chromium trichloride at altering lipid metabolism. These data of Cr can significantly alter lipid metabolism in diabetic rats were consistent with results published by Jain et al [35] and Sahin et al [36]. They found that supplementation with chromium picolinate at a dose of 80 or 400 μg/ Cr kg bw can significantly alter TC and TG levels in diabetic rats.

### Cr content analysis

The content of Cr in serum, heart, liver, spleen, lung, kidney and brain is shown in Table 2. The serum Cr content of type 2 diabetic rats is significantly lower than in normal rats. However, there are no significant decrease in the Cr content of heart, liver, spleen, lung, kidney and brain. This finding illustrates that T2D can reduce Cr content in serum. Chromium malate, chromium picolinate and chromium trichloride group can increase the Cr content in serum and organs. The serum Cr content of chromium malate and chromium picolinate treated groups is significantly higher than that of the model group. As seen from Table 2, the serum Cr content of chromium trichloride group was significantly lower than the Cr content of the chromium malate and chromium picolinate treated groups. This outcome illustrated that the chromium malate and chromium picolinate absorption was higher than that of the chromium trichloride group and the absorption rate of chromium malate was the same as that of chromium picolinate. The amount of Cr in rat serum is higher than in organs. It can be observed that the highest Cr content within the high dose group is found in the spleen, followed by—in descending order—the lungs, kidneys, brain, heart, and liver.

**Table 2:** Effects of chromium malate (Cr content) on the biochemical changes in the serum and organs of normal rats and type 2 diabetic rats.

Group	Parameter(μg/mL)	(μg/g)					
	Serum	Heart	Liver	Spleen	Lung	Kidney	Brain
**Normal control group**	15.67±1.53	6.95±1.13	6.09±4.18	10.59±0.58	14.98±0.84	9.49±0.59	10.07±4.31
**Model group**	10.90±1.55^a^	5.98±0.97	5.90±0.54	10.40±1.37	13.86±4.26	8.77±1.42	7.73±3.37
**Chromium picolinate group**	16.12±0.83^b^	7.35±1.58	6.19±4.43	15.09±1.25	15.09±2.24	10.64±0.83	12.69±3.31
**Chromium trichloride group**	12.00±1.00^a^ ^c^	5.96±2.70	5.96±2.02	13.15±7.35	14.09±1.13	8.96±2.12	11.85±1.37
**Chromium malate group (Low dose group)**	11.23±1.36^a^ ^c^	5.17±1.74	5.99±4.02	10.61±0.56	13.81±1.15	8.62±0.70	10.11±1.24
**Chromium malate group (Middle dose group)**	16.66±0.57^b^ ^d^	6.33±1.53	6.19±1.62	13.15±4.51	14.30±2.21	8.69±3.23	12.24±0.96
**Chromium malate group (High dose group)**	17.29±1.59^b^ ^d^	7.94±3.43	6.21±2.65	15.97±0.95	14.86±5.00	11.61±1.08	12.17±1.43
**Chromium malate control group**	15.66±0.57^b^ ^d^	7.19±2.46	6.16±2.31	11.40±7.97	14.73±1.53	10.07±1.79	11.68±5.70

Chromium picolinate and chromium trichloride was used as a positive control. Each value was presented as means±SD (n = 10).

^a^ Significantly different from normal control group (P < 0.05).

^b^ Significantly different from model group (P < 0.05).

^c^ Significantly different from chromium picolinate group (P < 0.05).

^d^ Significantly different from chromium trichloride group (P < 0.05).

### Effect of chromium malate on Body Mass changes in rats

The body mass changes of normal rats and type 2 diabetic rats treated with chromium malate are shown in the S2 Fig. It can be observed that the body mass of rats increased with an increase in feeding time in the normal control group, chromium picolinate group, low dose group, middle dose group, high dose group of chromium malate control group in the third and fourth weeks. The type 2 diabetic rats, which were fed with chromium malate, showed signs of body mass gain. There was a significant increase in the high dose group when compared with the model group. However, the body mass level of the chromium picolinate group and the chromium trichloride group did not increase significantly when compared with the model group.

### Histopathological analyses

**Fig 6:**
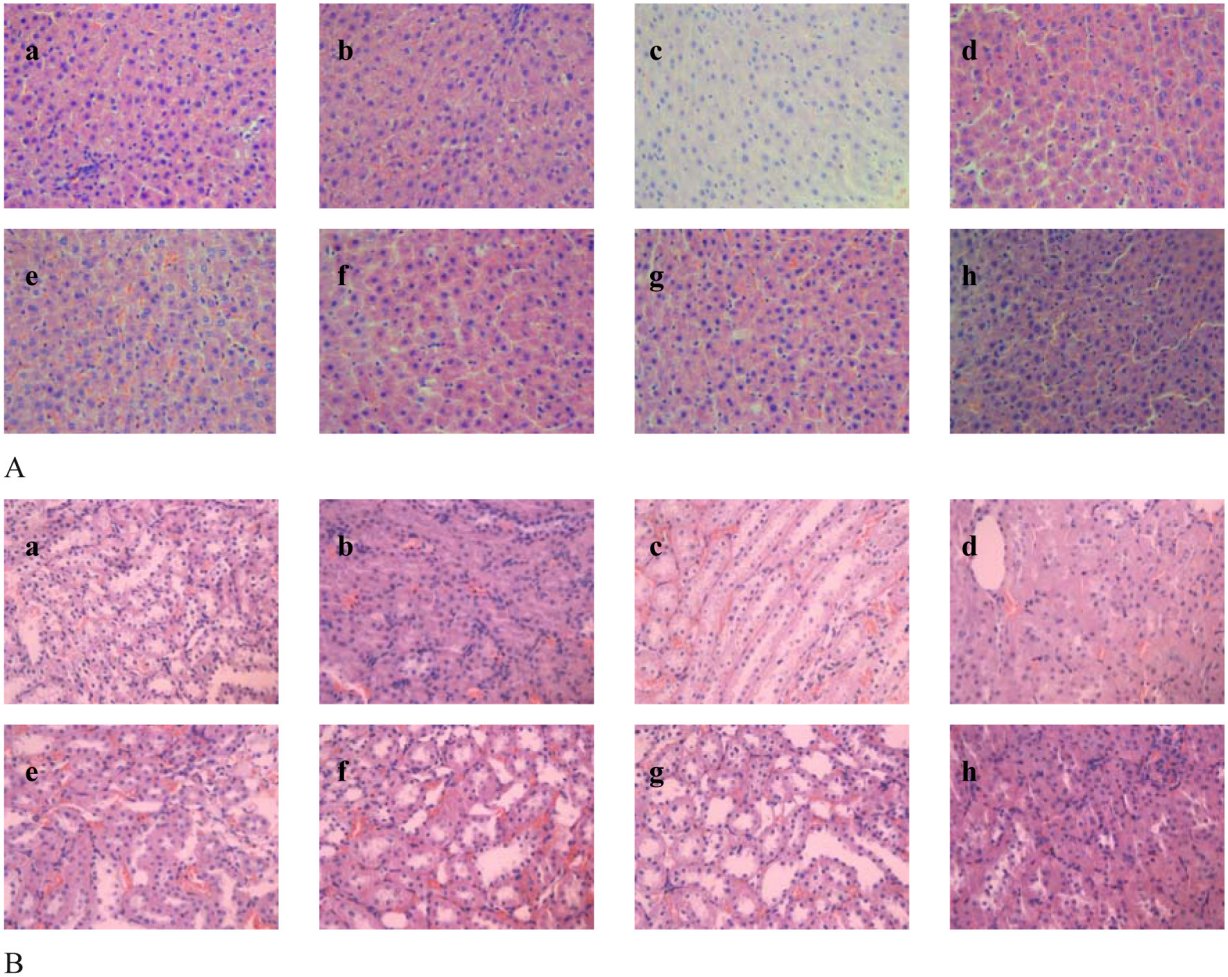
Effects of chromium malate on liver (A) and kidney (B) microanatomical features changes of the normal rats and type 2 diabetic rats. Chromium picolinate and chromium trichloride was used as a positive control. a–h represents Normal control group, Model group, Chromium picolinate group, Chromium trichloride group, Low dose group, Middle dose group, High dose group, chromium malate control group, respectively.

The liver and kidney microanatomical features changes of the normal rats and type 2 diabetic rats are shown in Fig 6. Cell necrosis, cell nucleus appeared cracking or dissolved and cell swell were not found in this study. The intact cells of liver, brain, kidney and testis/uterus can be found in this study. It can be observed that chromium malate has no-measurable damage to liver and kidney. Acute toxicities of chromium malate were tested by Wu et al [27] at a dose of 2.42 g Cr /kg with a single oral gavage. The results shown that chromium malate has no-measurable toxicity and consistent with ours.

### Effects of chromium malate on relative organ weight in rats

The relative organ weights of the heart, liver, spleen, lung, kidney and brain in chromium malate, chromium trichloride and chromium picolinate-treated rats are shown in the S1 Table. Chromium malate, chromium picolinate and chromium trichloride did not significantly alter relative organ weight when compared to the model group. The results indicated that chromium malate has no effect on the relative organ weight of type 2 diabetic rats. Li et al and Staniek et al [28,37] have also reported that chromium malate does not significantly affect the relative organ weight in diabetic rats and consistent with ours.

### The hematological and biochemical analysis

The biochemical and hematological analyses of normal rats and type 2 diabetic rats following administration of chromium malate, chromium picolinate and chromium trichloride by oral gavage are indicated in the S2 and S3 Tables, respectively. The biochemical indicators of chromium malate group did not change significantly when compared with the normal control group and the model group. These biochemical indicators are an important index of liver function. The results of hematological analyses are similar to those of our biochemical analyses (S3 Table). These results showed that chromium malate did not markedly affect biochemical and hematological indicators.

### Learning and memory ability and related enzymes study

#### Morris water maze test

**Fig 7:**
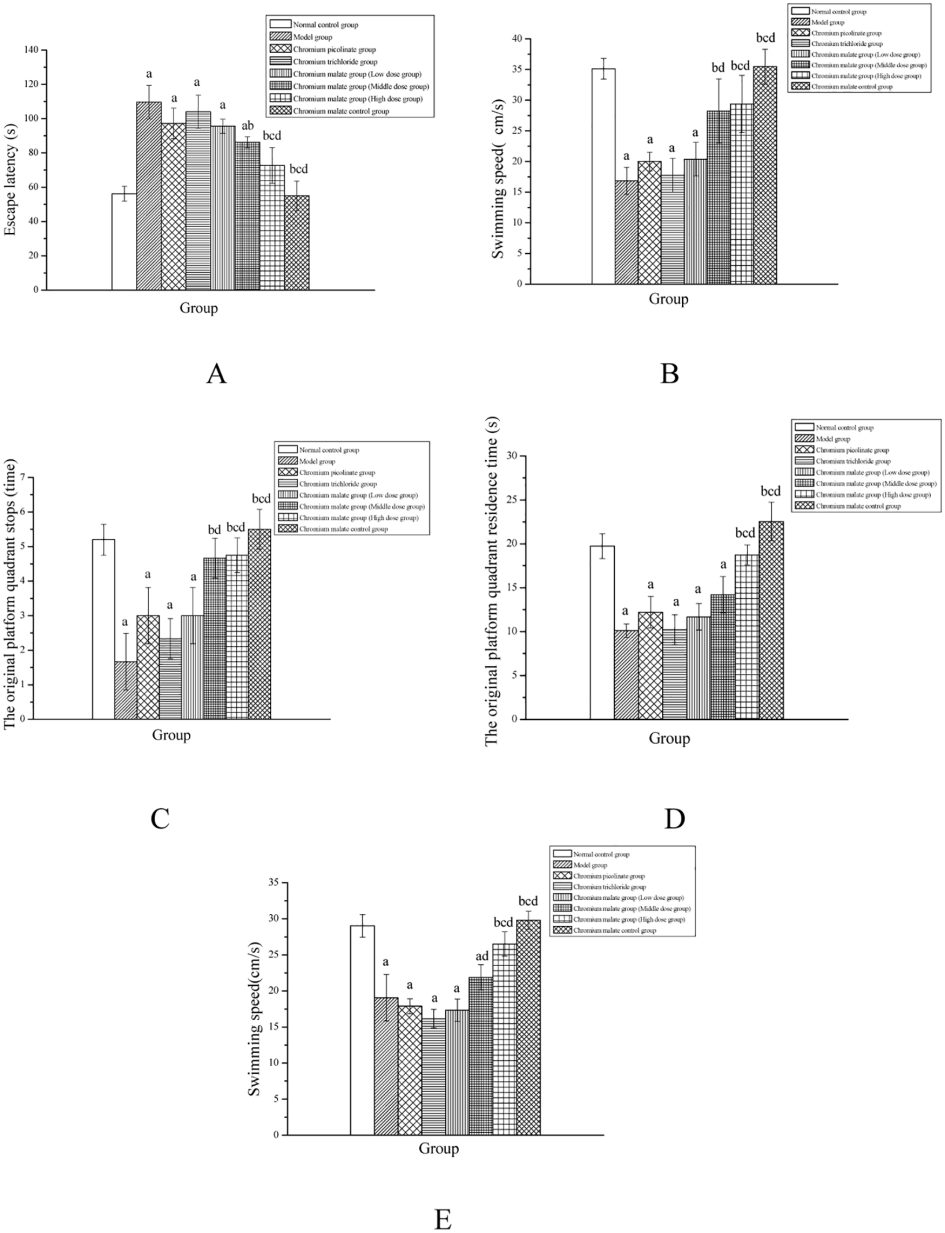
Effects of chromium malate on escape latency (A) and swimming speed (B) changes in the Morris water maze test. The original platform quadrant stops (A), residence time (B) and swimming speed (C) changes in space exploration experiment was tested at the same time. Chromium picolinate and chromium trichloride was used as a positive control. Each value was presented as means±SD (n = 10). ^a^ Significantly different from normal control group (P < 0.05). ^b^ Significantly different from model group (P < 0.05). ^c^ Significantly different from chromium picolinate group (P < 0.05). ^d^ Significantly different from chromium trichloride group (P < 0.05).

In this study, the normal rats and type 2 diabetic rats were subjected to the morris water maze test. The escape latency of type 2 diabetic rats was longer than that of normal rats, whereas their swimming speed was slower than that of normal rats (Fig 7A and 7B). Reports in the literature have indicated that T2D can affect learning and memory functions of rats [38,39]. Chromium malate (at a dose of 15.0 and 20.0 μg Cr/kg bw) can significantly decrease escape latency and increase swimming speed in type 2 diabetic rats. In contrast, treatment with chromium picolinate and chromium trichloride generated no significant change in these two indicators. The data of the high dose group changed significantly when compared with the model, chromium picolinate and chromium trichloride groups. The space exploration experiment of the normal rats and type 2 diabetic rats is shown in Fig 7C, 7D and 7E. Chromium malate (dose 20.0 μg Cr/kg bw) can significantly increase the original platform quadrant stops, residence time and swimming speed in type 2 diabetic rats. The results differed significantly when compared with the model, chromium picolinate and chromium trichloride groups. The curative effects of chromium malate on learning and memory ability were better than those of chromium picolinate and chromium trichloride.

#### The related enzyme analysis

**Fig 8:**
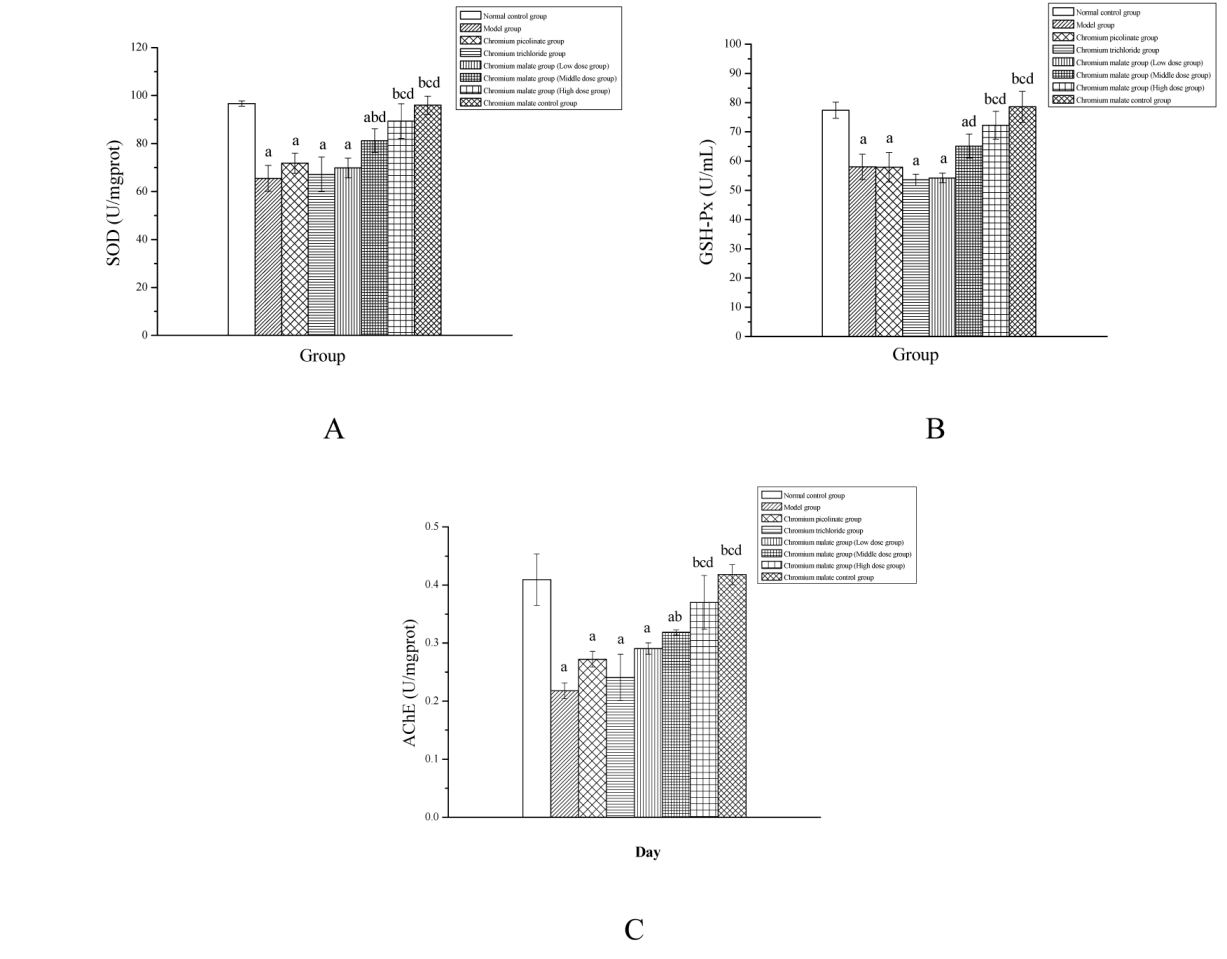
Effects of chromium malate on superoxide dismutase (SOD), glutathione peroxidase (GSH-Px) and true choline esterase (TChE) activity changes on hippocampus of normal rats and type 2 diabetic rats. Chromium picolinate and chromium trichloride was used as a positive control. Each value was presented as means±SD (n = 10). ^a^ Significantly different from normal control group (P < 0.05). ^b^ Significantly different from model group (P < 0.05). ^c^ Significantly different from chromium picolinate group (P < 0.05). ^d^ Significantly different from chromium trichloride group (P < 0.05).

Effects of chromium malate on SOD, GSH-Px and TChE activity in the hippocampus of normal rats and type 2 diabetic rats are shown in Fig 8. The activity of SOD, GSH-Px and TChE were found to be significantly decreased in type 2 diabetic rats compared to normal rats. Qiu et al [40] have shown that hippocampal damage will affect the learning and memory function of rats. Chromium malate and chromium picolinate can increase the activity of SOD, GSH-Px and TChE in type 2 diabetic rats. The data of the high dose group have a significant increase when compared with those of the model group, chromium picolinate group and chromium trichloride group. However, the activity of SOD, GSH-Px and TChE of the chromium picolinate group and chromium trichloride group has no significant increase when compared with the model group. The curative effects of chromium malate improve the activity of SOD, GSH-Px and TChE better than that of chromium picolinate and chromium trichloride.

### Effect of chromium malate on intestinal flora changes of rats

The intestinal flora changes of normal rats and type 2 diabetic rats following administration of chromium malate, chromium trichloride and chromium picolinate by an oral gavage are indicated in Table 3. Chromium malate, chromium picolinate and chromium trichloride can increase the number of G^+^b and Gb/Gc. The result of the high dose group was significantly higher than that of the model group, chromium picolinate group and chromium trichloride group. However, the data of the chromium trichloride group show no significant changes when compared with model group. At the same time, it can be observed that the number of G^+^b and Gb/Gc were decreased in type 2 diabetic rats and were significantly lower than that of normal rats. Many diseases can change the structure of the human intestinal bacteria [41], such as diabetes, autism, parkinson’s disease, multiple sclerosis, and rheumatoid arthritis. Therefore, it can be observed that the curative effects of chromium malate on improving the structure of intestinal flora are better than those of chromium picolinate and chromium trichloride.

**Table 3:** Effects of chromium malate on intestinal flora of normal rats and type 2 diabetic rats.

Group	Parameter				
	G^+^b	G^-^b	G^+^c	G^-^c	Gb/ Gc
**Normal control group**	94.87±3.27	101.92±5.11	0	3.21±0.46	61.31±4.15
**Model group**	37.91±3.51^a^	142.60±7.34^a^	1.79±0.14^a^	17.70±1.21^a^	9.26±1.79^a^
**Chromium picolinate group**	51.31±4.09^b^	134.01±5.57^a^	1.20±0.19^a^ ^b^	13.48±1.12^a^ ^b^	12.62±2.59^a^
**Chromium trichloride group**	46.56±2.73	136.85±6.87^a^	1.59±0.16^a^ ^c^	15.00±1.03^a^	11.06±2.59^a^ ^c^
**Chromium malate group (Low dose group)**	50.26±3.31^b^	134.45±6.03^a^	1.10±0.13^a^ ^b^ ^d^	14.19±0.97^a^ ^b^	12.08±1.86^a^ ^c^ ^d^
**Chromium malate group (Middle dose group)**	69.44±2.14^b^ ^d^	120.17±5.78^a^ ^b^ ^d^	0.52±0.08^a^ ^b^ ^c^ ^d^	9.87±1.44^a^ ^b^ ^c^ ^d^	18.25±1.26^a^ ^b^
**Chromium malate group (High dose group)**	81.46±3.84^b^ ^c^ ^d^	111.08±4.47^b^ ^c^ ^d^	0.33±0.08^b^ ^c^ ^d^	7.13±1.07^a^ ^b^ ^c^ ^d^	25.81±2.14^a^ ^b^ ^c^ ^d^
**Chromium malate control group**	92.07±2.27^b^ ^c^ ^d^	103.83±5.39^b^ ^c^ ^d^	0^b^ ^c^ ^d^	4.09±0.34^a^ ^b^ ^c^ ^d^	47.90±5.82^a^ ^b^ ^c^ ^d^

Chromium picolinate and chromium trichloride was used as a positive control. Each value was presented as means±SD (n = 10).

^a^ Significantly different from normal control group (P < 0.05).

^b^ Significantly different from model group (P < 0.05).

^c^ Significantly different from chromium picolinate group (P < 0.05).

^d^ Significantly different from chromium trichloride group (P < 0.05).

## Conclusion

Chromium malate exhibits greater benefits in treating type 2 diabetic-induced glycometabolism, glycometabolism-related enzyme activites and lipid metabolism disorders than chromium picolinate and chromium trichloride. The absorption rate of chromium malate and chromium picolinate is significantly higher than that of chromium trichloride. Chromium malate can significantly improve the learning and memory ability of T2D rats and the expression of related enzymes (SOD, GSH-PX and AChE), as well as the structure of intestinal flora, more efficiently than chromium picolinate and chromium trichloride. Therefore, it can be concluded that chromium malate can be used as an anti-diabetic functional food and nutritional supplement. The mechanisms of its anti-hyperglycemic activity, improvement of learning and memory capability and the structure of intestinal flora will be further investigated.

## Supporting Information

S1 Fig Fasting blood glucose (FBG) level—time curves. (A) and area under curves (AUC) (B) of FBG in normal rats and types 2 diabetic rats, 3 days after intraperitoneal injection of STZ. Data are expressed as means ± SD. Number 1: the AUC of 30min, Number 2: the AUC of 60min, Number 3: the AUC of 120min, Number 4: the AUC of 180min. *Significantly different from normal rats (P < 0.05).
(DOC)

S2 Fig The body mass (g) changes of normal and type 2 diabetic rats were treated with chromium malate. Chromium picolinate and chromium trichloride was used as a positive control. Each value was presented as means±SD (n = 10). ^a^ Significantly different from normal control group (P < 0.05). ^b^ Significantly different from model group (P < 0.05). ^c^ Significantly different from chromium picolinate group (P < 0.05). ^d^ Significantly different from chromium trichloride group (P < 0.05).
(DOC)

S1 File Ethics Statement. (DOC)

S1 Table Effects of chromium malate on relative organ weights changes of normal rats and type 2 diabetic rats. Chromium picolinate and chromium trichloride was used as a positive control. Each value was presented as means±SD (n = 10). ^a^ Significantly different from normal control group (P < 0.05). ^b^ Significantly different from model group (P < 0.05). ^c^ Significantly different from chromium picolinate group (P < 0.05). ^d^ Significantly different from chromium trichloride group (P < 0.05).
(XLS)

S2 Table Effects of chromium malate on the biochemical changes on serum of normal rats and type 2 diabetic rats. Chromium picolinate and chromium trichloride was used as a positive control. Each value was presented as means±SD (n = 10). ALT = alanine transaminase, AST = aspartate transaminase, ALP = alkaline phosphatase, TP = total protein, BUN = blood urea nitrogen, Crea = creatinine. ^a^ Significantly different from normal control group (P < 0.05). ^b^ Significantly different from model group (P < 0.05). ^c^ Significantly different from chromium picolinate group (P < 0.05). ^d^ Significantly different from chromium trichloride group (P < 0.05).
(XLS)

S3 Table Effects of chromium malate on the hematological changes on peripheral blood of normal rats and type 2 diabetic rats. Chromium picolinate and chromium trichloride was used as a positive control. Each value was presented as means±SD (n = 10). WBC = white blood cell, LYMPH = lymphocyte count, MNCC = mononuclear cell count, NCC = neutral cell count, RBC = red blood cell, HCT = hemataocrit, MCV = mean corpuscular volume, RDW = Red cell distribution width, Hb = hemoglobin, MHbC = mean hemoglobin content, HCT = hemataocrit, MCV = mean corpuscular volume, RDW = Red cell distribution width, Hb = hemoglobin, MHbC = mean hemoglobin content. ^a^ Significantly different from normal control group (P < 0.05). ^b^ Significantly different from model group (P < 0.05). ^c^ Significantly different from chromium picolinate group (P < 0.05). ^d^ Significantly different from chromium trichloride group (P < 0.05).
(XLS)
