# Supplementary material for: Type 2 Diabetic Rats on Diet Supplemented With Chromium Malate Show Improved Glycometabolism, Glycometabolism-Related Enzyme Levels and Lipid Metabolism
Source: PLoS One. 2015 May 5;10(5):e0125952. doi: 10.1371/journal.pone.0125952 (PMC4420285; doi:10.1371/journal.pone.0125952)
Supplement: S1 Fig — (A) and area under curves (AUC) (B) of FBG in normal rats and types 2 diabetic rats, 3 days after intraperitoneal injection of STZ. Data are expressed as means ± SD. Number 1: the AUC of 30min, Number 2: the AUC of 60min, Number 3: the AUC of 120min, Number 4: the AUC of 180min. *Significantly different from normal rats (P < 0.05). (DOC) [file pone.0125952.s001.doc]

A

**

**

**

**

B

**S1 Fig.**
