# Supplementary material for: Type 2 Diabetic Rats on Diet Supplemented With Chromium Malate Show Improved Glycometabolism, Glycometabolism-Related Enzyme Levels and Lipid Metabolism
Source: PLoS One. 2015 May 5;10(5):e0125952. doi: 10.1371/journal.pone.0125952 (PMC4420285; doi:10.1371/journal.pone.0125952)
Supplement: S2 Fig — Chromium picolinate and chromium trichloride was used as a positive control. Each value was presented as means±SD (n = 10). a Significantly different from normal control group (P < 0.05). b Significantly different from model group (P < 0.05). c Significantly different from chromium picolinate group (P < 0.05). d Significantly different from chromium trichloride group (P < 0.05). (DOC) [file pone.0125952.s002.doc]

**S2 Fig.**
