# Supplementary material for: Type 2 Diabetic Rats on Diet Supplemented With Chromium Malate Show Improved Glycometabolism, Glycometabolism-Related Enzyme Levels and Lipid Metabolism
Source: PLoS One. 2015 May 5;10(5):e0125952. doi: 10.1371/journal.pone.0125952 (PMC4420285; doi:10.1371/journal.pone.0125952)
Supplement: S1 File — (DOC) [file pone.0125952.s003.doc]

**Ethics Statement**

All the experimental procedures were conducted in accordance with The Code of Ethics of the World Medical Association (Declaration of Helsinki) for experiments involving humans，EC Directive 86/609/EEC for animal experiments, Uniform Requirements for manuscripts submitted to PLOS ONE, and approved by the Jiangsu University Committee on Animal Care and Use(the license number SYXK (SU) 2013–0036). Sprague-Dawley rats were procured from the Jiangsu University. Sprague-Dawley rat is not a protected nor endangered species. Our experiments complied with the laws and ethical recommendations currently in effect in China where the experiments were performed. The rats were anaesthetized by anhydrous diethyl ether in order to ameliorate suffering. Then the rats were sacrificed at unconscious situation.
